# Chinese Herbal Prescription Fu-Zheng-Qu-Xie Prevents Recurrence and Metastasis of Postoperative Early-Stage Lung Adenocarcinoma: A Prospective Cohort Study Followed with Potential Mechanism Exploration

**DOI:** 10.1155/2021/6673828

**Published:** 2021-05-12

**Authors:** Sufang Zhang, Wanqing Chen, Yuli Wang, Jianchun Wu, Lili Xu, Yongchun Yu, Jianhui Tian, Rongzhong Xu, Zhihong Fang, Lei Jiang, Yingbin Luo, Yan Li

**Affiliations:** ^1^Department of Oncology, Shanghai Municipal Hospital of Traditional Chinese Medicine, Shanghai University of Traditional Chinese Medicine, Shanghai, China; ^2^Department of Traditional Chinese and Western Medicine, Shanghai Pulmonary Hospital, Tongji University School of Medicine, Shanghai, China; ^3^Dean's Office, Shanghai Chest Hospital Affiliated to Shanghai Jiaotong University, Shanghai, China; ^4^Department of Oncology, Shanghai Longhua Hospital affiliated to Shanghai University of Traditional Chinese Medicine, Shanghai, China; ^5^Department of Thoracic surgery, Shanghai Pulmonary Hospital, Tongji University School of Medicine, Shanghai, China

## Abstract

Chinese herbal Fu-Zheng-Qu-Xie (FZQX) prescription has been found to improve the immune function and survival of patients with early-stage lung cancer. However, the therapeutic efficacy needs to be evaluated objectively, and the precise mechanism remains unclear. In the present study, a double-center, prospective cohort study was carried out to assess the clinical efficacy of the FZQX prescription in preventing the recurrence and metastasis of postoperative early-stage lung adenocarcinoma. Our results indicated that the FZQX prescription could significantly reduce the 3-year postoperative recurrence rate and improve the life quality. Moreover, the peripheral blood indices showed that the positive immune index (CD_4_^+^T/CD_8_^+^T) increased and the negative immune indices (CD_8_^+^T, Myeloid-derived suppressor cells (MDSCs), Treg) decreased after treatment with the FZQX prescription. Since the positive regulatory effect of the FZQX prescription on immune function, a series of experiments were conducted to verify the tumor-suppressive effect and elucidate the underlying mechanisms. Through the MDSC clearance xenograft model, we confirmed that the FZQX prescription could effectively suppress tumor growth with lesser side effects in vivo, and MDSCs may be involved in the biological process of the FZQX prescription's intervention in lung cancer progression. By establishing the coculture system of MDSCs/LLC to simulate the immune microenvironment of lung cancer, the tumor suppression effect of the FZQX prescription was further validated by in vitro experiments. Besides, it was confirmed that the FZQX prescription could regulate MDSCs to remodel the immunosuppressive tumor microenvironment, thus exerting its preventive effect on relapse of lung cancer. Finally, the pathway activator and inhibitor were further used to explore the potential molecular mechanism. Results demonstrated that the IL-1*β*/NF-*κ*B signaling pathway was one of the critical signaling pathways of FZQX prescription regulating MDSCs to prevent the recurrence and metastasis of lung adenocarcinoma.

## 1. Introduction

Because of its high incidence and mortality, lung cancer has been one of the leading causes of cancer-related death worldwide [1]. According to GLOBOCAN 2018, more than 2.1 million new lung cancers occurred and accounted for 1.8 million deaths per year [2]. Among males, China has one of the highest lung cancer incidence rates in the world. The incidence of lung cancer in the female is also at a high level [3]. Lung adenocarcinoma, as one of the most common histological types of lung cancer, accounts for 40% of total lung cancer cases [4]. For postoperative patients with early-stage lung adenocarcinoma, recurrence and metastasis are still the most important factors related to survival [5]. However, the use of postoperative adjuvant therapy for early-stage lung cancer remains controversial [6]. Although it was estimated that nearly a third of these patients would suffer local and distant recurrence in the future and the 5-year overall survival rate was only approximately 73% [7], there is not sufficient evidence regarding the effectiveness of adjuvant chemotherapy after surgery. Results of the JBR-10 clinical trial indicated that patients with a tumor diameter ≤ 4 mm could not benefit from adjuvant chemotherapy [8]. Considered the severe adverse reactions of chemotherapy ultimately, it is increasingly urgent to find an effective strategy to prolong the disease-free survival of these patients under the premise of life quality assurance.

In addition to surgery and chemotherapy, most Chinese patients seek help from traditional Chinese medicine (TCM) during or after standard first-line therapy. Numerous clinical trials have proved that TCM has its advantages in antitumors [9, 10]. The effects of TCM in alleviating symptoms, reducing side-effects, and improving quality of life and adverse events caused by chemotherapy have been confirmed [11, 12]. From the TCM point of view, the recurrence of lung cancer after surgery is closely related to the deficiency of both Qi and Yin on the one hand, as well as residual toxin and phlegm on the other. According to the philosophical theory in TCM, Qi is the most basic substance that makes up the human body and maintains the function of various organs. Yin has the function of nourishing and moisturizing organs. Both Qi and Yin are the material basis for the prevention of diseases. Varying degrees of Qi and Yin impairment were sustained after the surgery. However, there are still residual tumor cells that have not been completely removed. From the perspective of TCM, it is considered residual toxin and phlegm. Therefore, under the corresponding treatment principles of tonifying Qi and nourishing Yin as well as resolving phlegm and removing the toxin, the FZQX prescription was originated and applied in clinical for adjuvant treatment of lung cancer.

The FZQX prescription was composed of twelve commonly used herbs including *Astragalus membranaceus* (Fisch.) Bunge (Huang-Qi), *Glehnia littoralis* Fr. Schmidt (Bei-Sha-Sheng), *Ophiopogon Japonicus* (L.f) Ker-Gawl (Mai-Dong), *Atractylodes macrocephala* Koidz (Bai-Zhu), *Poria* cocos (Bai-Fu-Ling), *Codonopsis pilosula* (Franch.) Nannf. (Dang-Shen), *Selaginella doederleinii* Hieron (Shi-Shang-Bai), *Salvia chinensis* Benth (Shi-Jian-Chuan), *Solanum septemlobum* Bunge (Shu-Yang-Quan), *Prunella vulgaris* L. (Xia-Ku-Cao), *Sargassum pallidum* (Turn.) C.Ag. (Hai-Zao), and *Ostrea gigas* Thunberg (Mu-Li). Previous clinical study has revealed that the FZQX prescription could effectively reduce the levels of inflammatory factors (IL-6, TNF-*α*, IL-1*β*) and negative immune cells (Treg, MDSCs) in postoperative patients with early-stage lung adenocarcinoma and improve the quality of life [13]. However, limited by the short follow-up periods and inadequate sample size, the survival benefit was not significant.

In the present study, we conducted a double-center, prospective cohort study to further confirm the preventive efficacy of FZQX prescription on the recurrence of early-stage lung adenocarcinoma. Then, based on the influence of the FZQX prescription on the frequency of MDSCs in the peripheral blood, a series of in vivo and in vitro experiments were performed to explore the regulatory effect of the FZQX prescription on MDSCs. Finally, by verifying the related signaling pathway, we further elucidated the molecular mechanism of the FZQX prescription in inhibiting the progression of lung cancer by regulating MDSCs.

## 2. Methods

### 2.1. Patients and Clinical Data

From November 2016 to December 2018, a total of 481 participants following surgical resection for early-stage lung adenocarcinoma were recruited in the prospective cohort study. Taking FZQX prescription as the exposure factor, 240 participants in Shanghai Municipal Hospital of TCM were treated with the FZQX prescription and regular clinical follow-up (treatment group), whereas 241 patients in Shanghai Pulmonary Hospital were followed up regularly alone (control group). The major characteristics of each group of enrolled patients were listed in Table 1. The detailed inclusion and exclusion criteria were described in the Supplemental Methods. The study protocol was approved by the Regional Ethics Committee (Shanghai Municipal Hospital of TCM). All patients signed the informed consent, and this study was performed following the declaration of Helsinki. The clinical trial was registered at the Chinese Clinical Trial Registry (ChiCTR) with the registration number ChiCTR-POC-16009499.

The patients in the treatment group will receive the intervention of the FZQX prescription. The FZQX prescription was comprised of twelve commonly used herbs with various dosages. The specific drug composition, medicinal parts, and dosage were listed in Table 2. The FZQX granules were all purchased from Jiangyin Tianjiang Pharmaceutical Co. Ltd. of China. Fully dissolve the granules by adding 400 mL boiling water. Patients were required to take it twice daily in the morning and evening one hour after the meal. The total period of taking the drug should not be less than six months. The patients in the control group only received regular follow-up and were not allowed to take any other antitumor-related treatment, including chemotherapy, radiotherapy, and targeted therapy. All patients underwent routinely radiological imaging evolution consisting of computed tomography (CT), magnetic resonance imaging (MRI), and bone scan semiannually. Before and after six months of treatment or follow-up, all patients were observed for immune-related indicators (CD_4_^+^T, CD_8_^+^T, CD_4_^+^T/CD_8_^+^T, MDSCs, Treg) and Karnofsky Performance Score (KPS). Patients were also asked to fill out QoL questionnaires (EORTC QLQ-LC43) to assess the quality of life during the observation period. Besides, a cohort of healthy volunteers was also recruited. Levels of their peripheral immune cells were set as a reference. The baseline characteristics of the healthy control population were also listed in Table 1.

Patients were followed up carefully in the outpatient clinic or by telephone at least once six months until April 2020. Survival outcomes and other basic information of patients have mainly been obtained from electronic medical records in the hospital or telephone follow-up. Since the high 5-year overall survival rate of patients with early-stage lung adenocarcinoma, the primary outcome of this study was set as DFS. DFS was defined as the time from the date of study initiation to the date of either local recurrence or distant metastasis. Once recurrence or distant metastasis occurred, the patients were immediately withdrawn from the study. They would receive standardized treatment according to the NCCN guideline. Patients without recurrence or death were censored on the date of the last follow-up. Lose-to-follow was defined as the circumstance that patients have no clinic visit for three months or more. Patients lose-to-follow were excluded from the final analysis.

The sample size calculation was based on the 3-year DFS rate in our preliminary clinical trial. The expected 3-year DFS rate was 96.7% in patients treated with the FZQX prescription and 87.5% in the follow-up group. Set the alpha error probability to 0.05 and the power (1-*β*) to 0.9. The required sample size of each group was calculated to be 176. Considering a nonavailable and nonresponse rate of 20%, the desired sample size was 220 in each group. Due to the loss of follow-up, 16 patients in the treatment group were lost to follow-up during the clinical trial. Therefore, 465 patients (241 patients in the control group and 224 patients in the treatment group) were finally included and analyzed in this study. The flow chart describing enrollment, screening, grouping, and follow-up results of the clinical trial was shown in Figure S1.

### 2.2. The Components of the FZQX Prescription Qualified by UPLC-QTOF-MS

The UPLC-QTOF-MS analysis of the FZQX prescription was performed on Waters A CQUITY I-Class UPLC (Waters, Milford, MA, USA), which was equipped with a binary solvent manager, a sample manager, and a column manager. A Waters HSS T3 column (2.1 × 150 mm, 1.7 𝜇m) together with a Waters online filtrate 35°C was used. The mobile phase consisted of acetonitrile (B) and water containing 0.1% formic acid (*v*/*v*) (A) following a gradient elution program: 0–2 min: 0%–2% (B); 2–22 min: 2%–60% (B); 22–24 min: 60%–90% (B); 24–29 min: 90% (B); 29–30 min: 90%-0% (B); 30–35 min: 0% (B). The flow rate was set at 0.4 mL/min. 2 𝜇L of the test solution was injected for UPLC-QTOF-MS analysis. Data acquisition was controlled by MassLynx V4.1 software (Waters Corporation, Milford, USA). Automatic metabolite characterization was performed using UNIFI 1.8 (Waters, Milford, USA) by the search of the TCM library. The main components of the FZQX were listed in Figure S2 and Table S1.

### 2.3. Cell Lines and Cell Culture

The murine Lewis lung carcinoma (LLC) and A549 cell line were obtained from the cell bank of Shanghai Cell Bank of the Chinese Academy of Sciences. The LLC/A549 cells were cultured in Dulbecco's modified eagle medium (DMEM) with 10% fetal bovine serum (FBS) and 0.1% penicillin-streptomycin. Cells were incubated in a 37°C/5% CO2 atmosphere-designated incubator.

### 2.4. Reagents

The DMEM medium and 10% FBS were obtained from Gibco Life Technologies (Grand Island, NY, USA). Gemcitabine (GEM) was brought from Selleckchem. The PBS medium, Annexin V-FITC/PI apoptosis detection kit, and BCA protein assay kit were purchased from KeyGEN Biotech (Jiangsu, China). 5%BSA, paraformaldehyde (PFA), electrophoresis solution, transfer solution, CCK-8 kit, RIPA, and SDS-PAGE gel were all provided by Beyotime Biotech (Beijing, China). The mouse tumor and spleen dissociation kits were obtained from Miltenyi (MACS, Miltenyi Biotec, Germany). Anti-mouse PE-CD11b, anti-mouse FITC-Gr-1, anti-mouse PerCP/Cy5.5-Ly6C, anti-mouse APC-Ly6G, anti-mouse PE NKG2D, anti-mouse APC-CD49b, anti-mouse FITC-CD4, and anti-mouse APC-CD25 were purchased from BioLegend. RT-PCR primers were designed and provided by Sangon Biotech (Shanghai, China). Trizol reagent, Tween-20, and 20× TBS buffer were obtained from Thermo Scientific (Rockford, IL). An ECL Kit was provided by Tanon Biotech (Shanghai, China).

### 2.5. MDSCs Induction and Identification

MDSCs were induced in vitro. Firstly, the mice were sacrificed by cervical dislocation. Femurs and tibiae were harvested, and the bone marrow cavity was flushed with RPMI 1640 medium to collect the bone marrow cells. After filtering through a 30 *μ*m mesh filter, a cell suspension was obtained and centrifuged at 1000 RPM for 5 min. Then, discard the supernatant and add 5 mL erythrocyte lysate to the incubation to remove erythrocytes. Finally, GM-CSF (20 ng/mL) and IL-6 (20 ng/mL) were added and cultured in an incubator with 5% CO_2_ for the next 4 days and used for subsequent experiments. The purity of MDSCs was determined by flow cytometry. On the fourth day of culture, flow cytometry indicated that the proportion of PE-CD11b/FITC-Gr-1 double-positive cells was about 85.83 ± 7.54%, and the proportion of CD11b^+^Ly6C^+^Ly6G^−^ cells was about 47.73 ± 4.01%. RT-qPCR showed that after induction, the expression levels of Arg-1 and iNOS mRNA showed an increasing trend, which were the characteristic markers of MDSCs. Besides, other MDSCs-related cytokines such as IL-6, IL-10, and TGF-*β* were also highly expressed (as shown in Figure S3).

### 2.6. Cellular Function Assay

Cell proliferation was analyzed by the CCK8 assay. Log-phase LLC, A549 cells, and MDSCs cell suspension were collected and incubated in a 96-well plate (5 × 10^3^ cells per well). After 24 h of culture, 10 *μ*L CCK-8 reagent was added to each well, followed by cell incubation for 2 h at 37°C. Then, the optical density (OD) value was detected at a wavelength of 450 nm with a microplate reader. A colony formation assay was carried out as previously described to evaluate colony-forming ability [14]. LLC/A549 cells were inoculated in a 6-well plate with 500 cells per well. At 1 week, the cells were fixed with 4% PFA, and the number of single-cell clones was detected after crystal violet staining.

For wound healing assay, the treated LLC/A549 cells were inoculated on the 24-well plate at a density of 5 × 10^5^ cells/mL. When the cells formed monolayer cells, they were scratched with the tip of a 10 *μ*L pipette. The cells were washed three times with PBS and incubated for 24 h. The migration ability of the cells was observed under a microscope.

In the Transwell invasion assay, 100 *μ*L Matrigel was spread into the chamber, and the concentration of LLC cells was adjusted to 2 × 10^5^ cells/mL. Add 500 *μ*L complete medium containing 20%FBS to the 24-well plate. 100 *μ*L cell suspension was added in the Transwell chamber and incubated at 37°C for 24 h. After fixed and stained, the cells were counted under the microscope (magnification, ×200).

### 2.7. Establishment of the Conditional Medium

To investigate the effects of MDSCs on the proliferation, migration, invasion of tumor cells, and its impact on immune cells in the tumor microenvironment, a conditioned medium was generated from MDSCs treated with the FZQX prescription to simulate a tumor microenvironment. Firstly, based on the previous result of the concentration screening assay, the intervention was coped with 60 mg/mL of the FZQX prescription for 24 h. Then, the original medium was removed and the serum-free medium was added. After 12 h of incubation, the supernatant was collected as the conditional medium. The detailed steps were described in Figure S4.

### 2.8. Immunofluorescence

Log-phase LLC cells were collected and seeded in 24-well plates plated with cell-climbing slices (5 × 10^3^-1 × 10^4^ cells per well). After drug intervention for 24 h, the cells were fixed with 4% PFA for 15 min and then washed with PBS. Following blocking for 1 h at room temperature, the cells were incubated with primary antibodies (120 *μ*L per well) in blocking solution overnight at 4°C, followed by secondary antibody incubation at room temperature for 1 h. After washing with PBS and staining with DAPI, the slices were photographed (magnification, ×200) under a fluorescence microscope (Leica, Germany).

### 2.9. Immunohistochemical Staining

Tumor tissue was fixed with 4% PFA for 24 h, embedded with paraffin, and sectioned. After dewaxing and rehydration, the sections were boiled in citric acid antigen extract for 10 min. The primary antibody was incubated at 4°C, washed, and the secondary antibody was incubated at room temperature for 1 h. Fluorescence expression and localization were observed under a fluorescence microscope (magnification, ×200).

### 2.10. Drug Preparation

The granules of the FZQX prescription for in vivo experiments were purchased from Jiangyin Tianjiang Pharmaceutical Co. Ltd. of China. The equivalent dose of the drug was calculated according to the ratio of mice to the human body surface area of 1 : 4, and the concentration of the drug was calculated as 500 mg/mL, and the dose was 200 *μ*L for each mouse. Fully dissolve the granules in double-distilled water in a water bath pot at 80°C. Let the solution cool to room temperature and keep in the fridge at 4°C until use.

In in vitro experiments, the lyophilized powder of the FZQX prescription was prepared. All herbs were provided by the pharmacy of Shanghai Municipal Hospital of TCM and authenticated by Professor Haiqing Zhu. Firstly, the extract of the FZQX prescription was diluted 10 times in distilled water and heated for three hours under the condition of continuous stirring at 100°C. Repeat the process twice, and centrifuge the extract at 1500 g. The supernatant was collected and evaporated at 70°C until semisolid was formed. Triethanolamine was used as a neutralizer to regulate the pH value between 6 and 8 of the lyophilized powder. The mixture concentration was diluted to 1 g/mL by DMEM and stored at -20°C until use. The composition and quality control of the FZQX lyophilized powder was performed by UPLC/QTOF MS. A sample of the FZQX lyophilized powder was kept in our laboratory.

### 2.11. Establishment of Subcutaneous Tumor Xenograft Model

Male C57BL/6 mice (5~6 weeks old, 18~22 g) were purchased from Shanghai Slack Laboratory Animal Company (Shanghai, China) and maintained under SPF conditions, at 22 ± 2°C, a relative humidity of 55 ± 5%, and a 12 h/12 h light/dark cycle. To establish subcutaneous tumor-bearing mice, 2 × 10^6^ LLC cells suspended in 100 *μ*L PBS were injected into the right flank of the mice. The tumor-bearing mice were randomly assigned according to the purpose of the experiment. Each group of mice received the respective treatment by oral gavage or intraperitoneal injection for 21 days. The length and width of the subcutaneous tumors were measured, and the mice were weighed daily. At the end of the experiment, the mice were euthanized, and the xenograft tumors were isolated and weighed. All animal protocols were performed in compliance with the requirements of the Animal Ethics Committee of Shanghai Municipal Hospital of TCM (Shanghai, China).

### 2.12. RT-qPCR

Total RNA was isolated from cells or tissues using Trizol reagent (Thermo Scientific, Waltham, MA, USA) according to the manufacturer's instructions. 1 *μ*g of total RNA was reverse transcribed into cDNA using the reverse transcription kit (TaKaRa, Dalian, China) according to the manufacturer's protocols. The real-time quantitative PCR analysis was performed in a 20 *μ*L reaction carried out by SYBR Green PCR Mastermix (TaKaRa, Dalian, China). Related primers were listed in Table S2. Amplification of target genes was carried out to obtain the amplification curve and the melt curve of target genes according to the following steps: after 10 minutes of predenaturation step at 95°C, it then entered the denaturation-annealing-extension cycle. Among them, the denaturation step lasted for 10 seconds at 95°C, the annealing step lasted for 20 seconds at 60°C, the extension step lasted for 30 seconds at 72°C, and 40 cycles were carried out in total. The Ct value of each gene was detected and recorded. The expression level of the GAPDH gene was taken as the endogenous control. The method of 2^−ΔΔCt^ was used to calculate the relative gene expression levels.

### 2.13. Western Blotting

The tumor tissues and bone marrow cells were fully lysed with lysis buffer, and the supernatants were collected for quantitative protein analysis after centrifugation. A total of 50 *μ*g protein was added to each well of SDS-PAGE and transferred to polyvinylidene fluoride (PVDF) membranes through wet transfer. The PVDF membranes were blocked with 5% BSA for 2 h at room temperature. Then, incubation with the related primary antibodies was performed overnight at 4°C. The next day, the secondary antibody was added and incubated at room temperature for 1 h. The specific primary antibodies and secondary antibodies used in the present study were listed in Table S3. Finally, the protein bands were detected using an ECL detection kit (Tanon, Shanghai, China) and visualized using the BIO-RAD imaging system. The qualification of the protein bands was carried out with Image J software by densitometry.

### 2.14. Statistical Analysis

SPSS 25.0 for Windows (SPSS Inc, Chicago, IL, USA) and GraphPad Prism 8.0 (GraphPad Software Inc, California, USA) were adopted for performing statistical analyses and plotting. PASS 15.0 software (NCSS, Kaysville, Utah, USA) was used to calculate the sample size of the clinical trial. All data of continuous variables were presented as mean ± standard deviation (SD). Data conforming to a normal distribution were analyzed using either Student's *t*-test for two groups or one-way ANOVA for three or more groups. A nonparametric test was used for data of nonnormality. The counting data were presented as the rate (%) and analyzed with the Chi-square test. All statistical tests were two-tailed, and a *P* value of less than 0.05 was considered statistically significant.

## 3. Results

### 3.1. The FZQX Prescription Improved the Quality of Life, Immune Function, and the 3-Year DFS Rate of Postoperative Patients with Early Lung Adenocarcinoma

To ensure the quality and reliability of the clinical trial, we firstly compared the baseline characteristics of the patients between the two groups as well as healthy controls (as shown in Table 1). Results showed that there was no significant difference in the baseline data among the three groups, which indicated a comparable performance among groups.

In comparison with patients who received no FZQX prescription, patients treated with the FZQX prescription showed greater benefits in terms of life quality, as manifested by EORTC QLQ-LC43 functional score and KPS. From the EORTC QLQ-LC43 scale perspective, patients in both groups experienced various degrees of improvement in the quality of life after treatment or follow-up alone. However, the treatment group showed significantly better improvement in functional areas, specific symptoms, and general health (as shown in Tables 3(a)–3(e)). The improvement rate was defined as the ratio of patients who had a KPS score increase of >10. In contrast, a KPS score decrease of >10 was defined as deterioration. Our results showed that after intervention by the FZQX prescription, the KPS score (87.14 vs. 83.11) and the improvement rate (58.5% vs. 41.5%) of the patients in the treatment group were significantly higher than those in the control group (as shown in Tables 4 and 5).

For immune function, the levels of CD_8_^+^T, MDSCs, and Treg were significantly decreased in the treatment group. In contrast, the ratio of CD_4_^+^T/CD_8_^+^T increased in the treatment group. Furthermore, compared with the control group, the levels of immune function in the treatment group after treatment were closer to the healthy volunteers' group (as shown in Tables 6(a) and 6(b)). This provided evidence for the role of the FZQX prescription in regulating the immune function of postoperative patients with early-stage lung adenocarcinoma.

**Figure 1:**
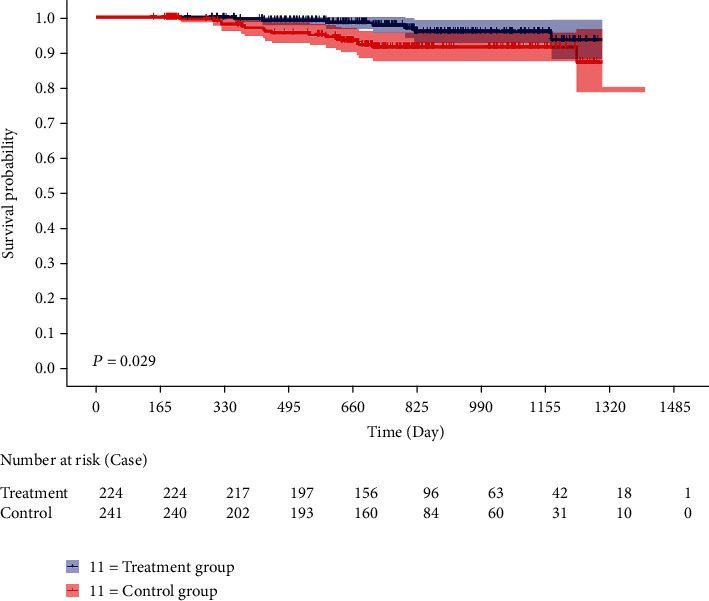
The DFS survival curve of the two groups. Blue curve represented for the treatment group, and red curve represented for the control group.

Finally, survival curves were plotted using the method of Kaplan-Meier to compare the DFS between the two groups. The survival curves were compared by the Log-rank test. Results showed that as of the data cutoff date, a total of seven patients (3.1%) in the treatment group exhibited recurrence compared with seventeen (7.1%) in the control group. The 1-year, 2-year, and 3-year DFS rates of the treatment group were higher than those in the control group (99.1%, 97.0%, and 93.7% vs. 97.6%, 91.7%, and 87.5%), which indicated that patients intervened by the FZQX prescription had an obvious improvement in DFS rate (as shown in Table 7 and Figure 1).

In summary, the clinical outcomes indicated that patients intervened by the FZQX prescription sustained a better-improved life quality, positive regulation of immune function, and a significant DFS benefit.

### 3.2. The FZQX Prescription Inhibited the Growth of Subcutaneous Xenograft of LLC Cells In Vivo

**Figure 2:**
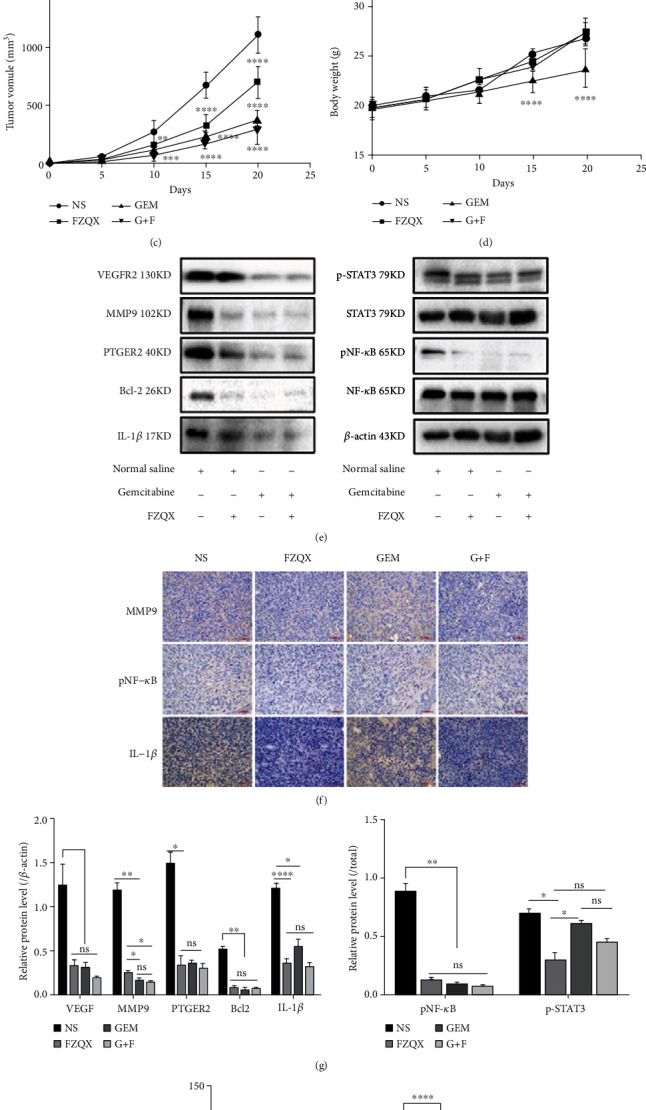
The FZQX prescription inhibited the growth of subcutaneous xenograft of LLC cells in vivo. (a) Representative photographs of xenograft tumors were taken 21 days at the experimental endpoint. (b) Weight of the xenograft tumors harvested from mice in each group. (c) The volume of the xenograft tumors harvested from mice in each group. (d) Dynamic change in the bodyweight of mice in each group. (e) Expression levels of proteins associated with MDSC immunosuppression function and tumor progression in each group detected by Western blot. (f) Expression levels of MMP9, pNF-*κ*B, and IL-1*β* proteins in the tumor tissues analyzed by immunohistochemistry. (g) Densitometric analysis of Western blot. (h) The signal intensity of immunofluorescent images quantified by ImageJ software. NS: the normal saline group (control); F: the FZQX prescription group; GEM: the gemcitabine group; G+F: the gemcitabine plus FZQX group. All data are expressed as the mean ± SD from at least three independent experiments. ns: no significant difference, ^∗^*P* < 0.05, ^∗∗^*P* < 0.01, ^∗∗∗^*P* < 0.001, ^∗∗∗∗^*P* < 0.0001. The same as below.

Since we found that the FZQX prescription reduced the frequency of MDSCs in the peripheral blood of patients with lung cancer in our preliminary clinical study, we further investigate whether MDSC-exhausted contributed to the ability of FZQX prescription to inhibit LLC growth in vivo. We established a xenograft model by subcutaneously injecting LLC cells in the C57BL/6 mice. Gemcitabine, as a traditional chemotherapeutic drug, has been reported to have selective cytotoxicity against MDSCs and could be used to reduce the number of MDSCs in animal models [15, 16]. Therefore, it was applied to establish the MDSC clearance xenograft model in the present study. Mice were randomly separated into four groups, including the normal saline group (NS), the FZQX prescription group (FZQX), the gemcitabine group (GEM), and the FZQX prescription plus gemcitabine group (G+F), respectively. The FZQX prescription was administered by gavage, while gemcitabine (10 mg/kg) was given via peritoneal injection. The tumor volume and body weight of the mice were monitored throughout the experiment. Results showed that in the GEM group, the tumor volume and body weight were both significantly reduced. Similar results were also seen in the FZQX group and the G+F group. Compared with the GEM group, the tumor-free weights of mice in the FZQX group and the G+F group were higher, which indicated that the FZQX prescription could effectively suppress tumor growth with lesser side effects (as shown in Figures 2(a)–2(d) and Table 8). Furthermore, the expression levels of proteins associated with MDSCs immunosuppression function and tumor progression were detected by Western blot. Results showed that the expression levels of MMP9, PTGER2, IL-1*β*, p-STAT3, Bcl-2, and pNF-*κ*B proteins were significantly decreased in the three intervention groups compared with the control group (Figure 2(e)). These results were also consistent with the immunofluorescence analyses (Figure 2(f)). The above experimental results confirmed the tumor-suppressive effect of the FZQX prescription in vivo, and its efficacy was comparable to that of the MDSC clearance model. Base on this, we speculated that MDSCs may be involved in the biological process of the FZQX prescription's intervention in lung cancer progression.

### 3.3. MDSCs Played a Key Role in the Inhibition of Tumor Progression by the FZQX Prescription

**Figure 3:**
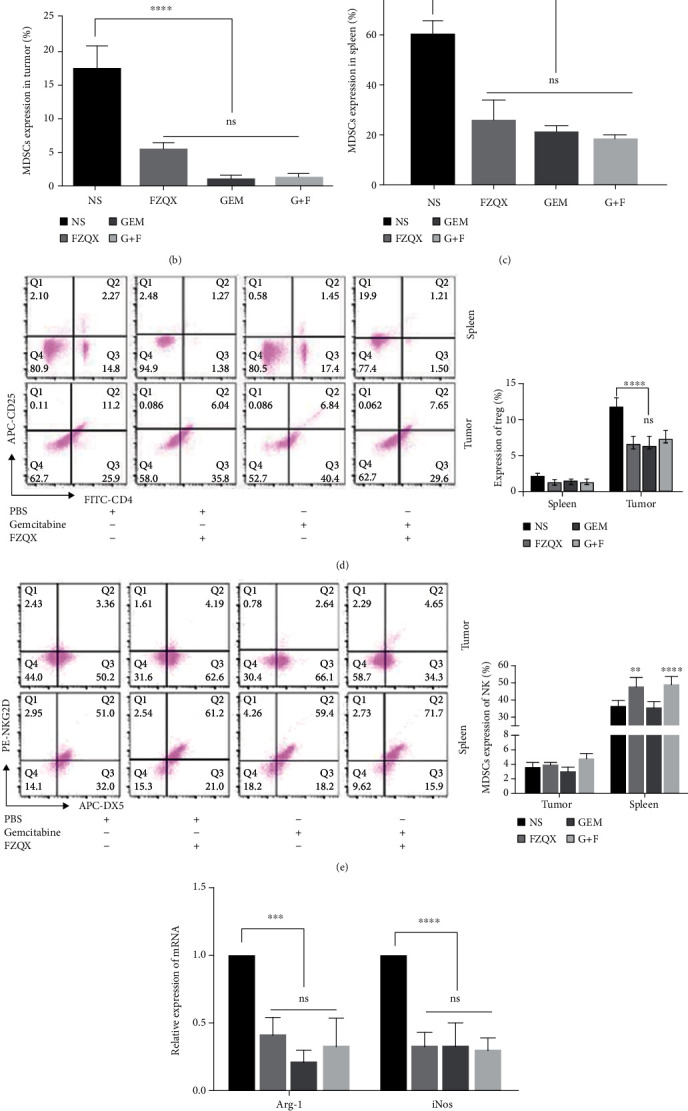
MDSCs played a key role in the inhibition of tumor progression by the FZQX prescription. Flow cytometry was performed on the percentage of MDSCs, Treg, and NK in spleens and tumor tissues. (a–c) The proportion of CD11b^+^Gr-1^+^ cells in the tumor tissues and spleens was determined (*n* = 4). (d) The proportion of CD_4_^+^CD_25_^+^ cells in the tumor tissues and spleens was determined (*n* = 4). (e) The percentage of Dx5^+^NKG2D^+^ cells in the tumor tissues and spleens was analyzed (*n* = 4). (f) Arg-1 and iNOS mRNA expression levels were quantified by RT-qPCR. (g) The concentration of serum IL-1*β* and CCL4 were measured by ELISA.

To further explore the role of MDSCs in the tumor immune microenvironment, we detected the infiltration of immune cells in spleen and tumor tissues. At the experimental endpoint on day 21, the expression levels of MDSCs, Treg, and NK cells in the tumor tissues and spleens were detected by flow cytometry. Results indicated that in the GEM group, the infiltrations of MDSCs and Treg in the tumor tissues and the spleen of mice were reduced. Similar results were also seen in the FZQX group and the G+F group. No significant differences were observed among the three intervention groups. Besides, compared with the control group and the GEM group, both the FZQX group and the G+F group could promote the expression of the NKG2D receptor in spleen tissues (as shown in Figures 3(a)–3(e)). Results of RT-qPCR showed a significant downregulation in the expression of Arg-1 and iNOS mRNA in the tumor tissues of the three intervention groups, compared with the NS group (Figure 3(f)). The ELISA results demonstrated that the concentration of serum IL-1*β* was significantly decreased in the three intervention groups. Besides, the concentration of serum CCL4 showed a marked decline in the G+F group, compared with the control group (Figure 3(g)). These results confirmed that MDSCs were one of the critical cell populations for the FZQX prescription to prevent the recurrence and metastasis of lung cancer.

### 3.4. The FZQX Prescription Suppressed the LLC/A549 Proliferation, Migration, and Invasion by Regulating MDSCs

**Figure 4:**
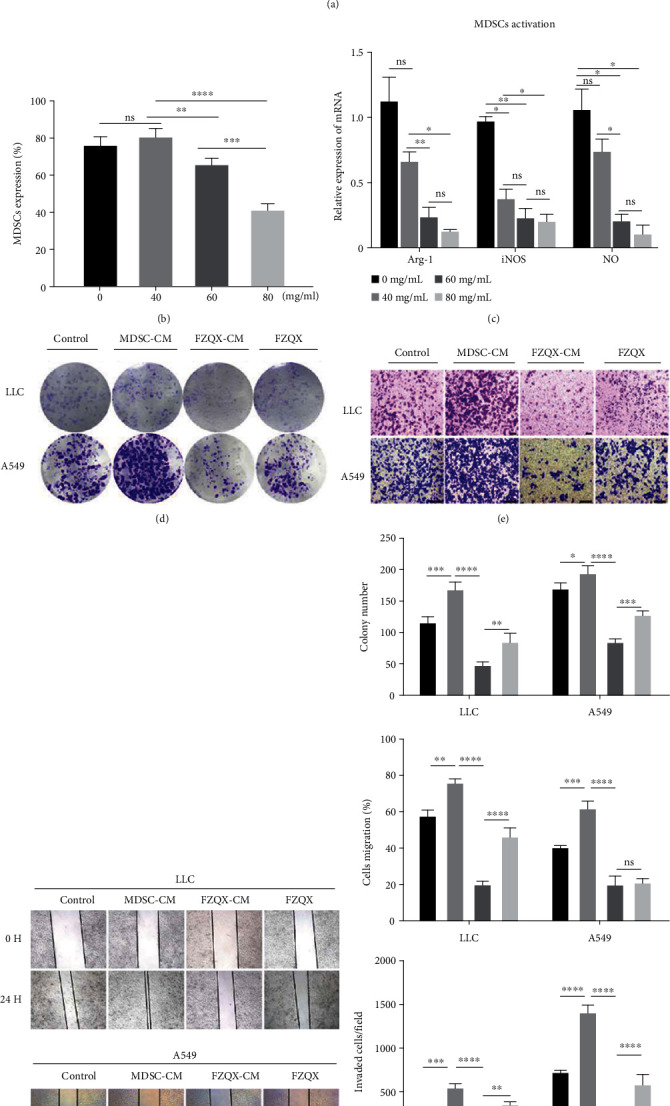
The FZQX prescription suppressed the LLC/A549 proliferation, migration, and invasion by regulating MDSCs. (a, b) The frequencies of MDSCs in different drug concentration gradients were determined through flow cytometry analysis. (c) MDSC activation-related mRNA expression levels were determined by RT-qPCR. (d) LLC/A549 cell proliferation was detected by colony formation assay. (e) LLC/A549 cell invasion was detected by Transwell assay. (f) LLC/A549 cell migration was examined by wound healing assay. (g) Quantification of the colony formation assay, the Transwell assay, and the wound healing assay results determined by ImageJ software. Control: LLC/A549 cell with no intervention; MDSC-CM: LLC/A549 treated with conditional medium (CM) derived from MDSCs culture; FZQX-CM: LLC/A549 treated with CM derived from the FZQX prescription culture; FZQX: LLC/A549 treated with the FZQX prescription.

To ascertain the appropriate concentration for subsequent in vitro experiments, the inhibitory effects of the FZQX prescription on MDSCs and LLC/A549 cells were determined by the CCK-8 assay. Results demonstrated that the half-maximal inhibitory concentration (IC50) values of the FZQX prescription for the MDSCs, LLC cells, and A549 cells were 72.69 mg/mL, 40.74 mg/mL, and 124.21 mg/mL, respectively. Thus, the concentration of the FZQX prescription was chosen as 60 mg/mL, and three concentrations of 40 mg/mL, 60 mg/mL, and 80 mg/mL were selected for the subsequent experiments (as shown in Figure S5). The flow cytometry analysis indicated that the FZQX prescription significantly reduced the frequency of MDSCs in a dose-dependent manner (Figures 4(a) and 4(b)). Meanwhile, the mRNA levels of MDSC activation-related genes showed a substantial decrease with increasing concentration of the FZQX prescription (Figure 4(c)).

Since MDSCs play a critical role in promoting tumor immune escape in the tumor microenvironment, we next established the coculture system of LLC/A549 and MDSCs in vitro to simulate a tumor microenvironment. Results of the colony formation assay, the wound healing assay, and the Transwell invasion assay indicated that the MDSCs-CM group could significantly promote cell proliferation, migration, and invasion of the LLC/A549. On the contrary, the cell proliferation, migration, and invasion activity were significantly inhibited in the FZQX-CM group, which showed an obvious difference from the group intervened by the FZQX prescription alone (FZQX group) (as shown in Figures 4(d)–4(f)). The above functional experiments demonstrated that the FZQX prescription could suppress the proliferation, migration, and invasion of LLC/A549 by regulating the frequency and function of MDSCs.

### 3.5. The FZQX Prescription Suppressed the Activation IL-1*β*/NF-*κ*B Signaling Pathway in MDSCs

**Figure 5:**
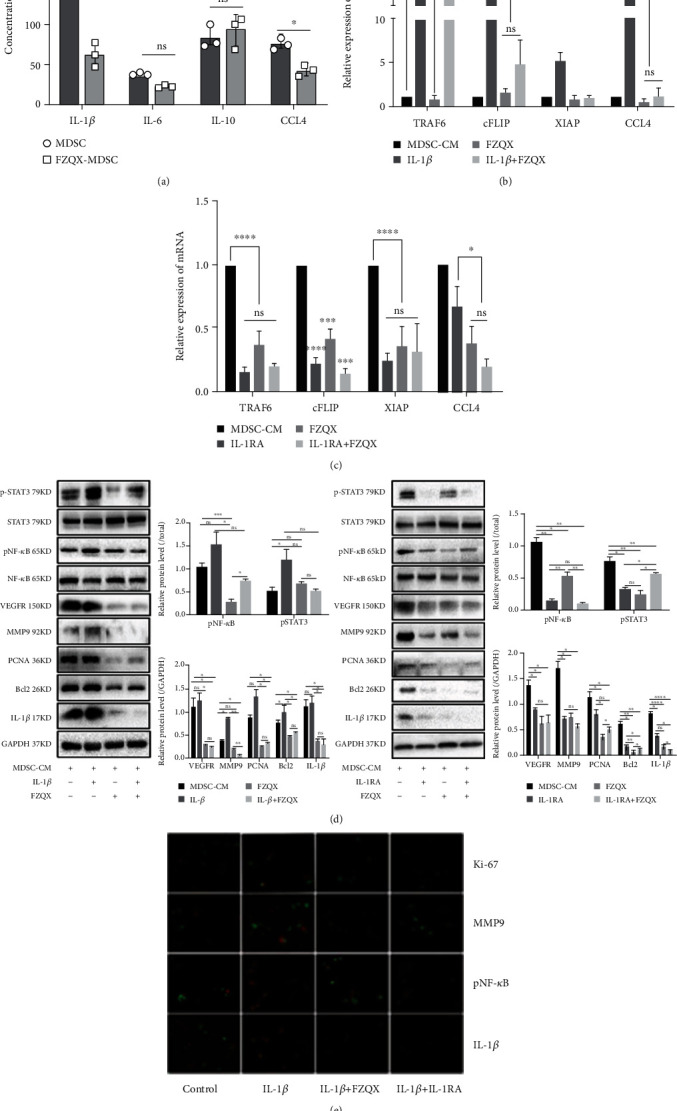
The FZQX prescription suppressed the activation IL-1*β*/NF-*κ*B signaling pathway in MDSCs. (a) Culture supernatants were collected and analyzed for cytokine concentration by ELISA. (b, c) The mRNA expression levels of IL-1*β*/NF-*κ*B signaling pathway-related genes were determined by RT-qPCR, including TRAF6, cFLIP, XIAP, and CCL4. (d) Western blot detected the expression levels of key proteins related to tumor proliferation, invasion, and metastasis involved in the IL-1*β*/NF-*κ*B signaling pathway, including VEGFR, MMP9, PCNA, and involved in signaling pathways, including Bcl2, IL-1*β*, NF-*κ*B, and STAT3. (e) Immunofluorescence detected the expression levels of Ki-67, MMP9, pNF-*κ*B, and IL-1*β*. Control (MDSCs-CM): MDSCs conditional medium; FZQX: MDSCs-CM intervened by the FZQX prescription; IL-1*β*: MDSCs-CM intervened by IL-1*β*; IL-1RA: MDSCs-CM intervened by IL-1 receptor antagonist; IL-1*β*+FZQX: MDSCs-CM intervened by IL-1*β* and the FZQX prescription simultaneously; IL-1RA+FZQX: MDSCs-CM intervened by IL-1RA and the FZQX prescription simultaneously. IL-1*β*+ IL-1RA: MDSCs-CM intervened by IL-1*β* and IL-1RA.

To explore the potential mechanism of the FZQX prescription's regulation of MDSCs in the intervention of lung cancer progress, the culture supernatants of MDSCs were collected and evaluated for cytokine expression by ELISA. Results indicated that after intervention with the FZQX prescription, the level of IL-1*β* and CCL4 decreased significantly while other cytokines did not show any significant changes (as shown in Figure 5(a)). Among them, the decreasing trend of IL-1*β* was the most obvious. Therefore, to further verify the critical role of the IL-1*β* in the biological process of MDSCs induced lung cancer progression intervened by the FZQX prescription, we applied activator and inhibitor of IL-1*β* to set up the following groups, including the control group (MDSCs-CM), the IL-1*β* group (MDSCs-CM intervened by IL-1*β*), the FZQX group (MDSCs-CM intervened by the FZQX prescription), the IL-1 receptor antagonist (IL-1RA) group (MDSCs-CM intervened by IL-1RA), the IL-1*β*+FZQX group (MDSCs-CM intervened by IL-1*β* and the FZQX prescription simultaneously), and the IL-1RA+FZQX group (MDSCs-CM intervened by IL-1RA and the FZQX prescription simultaneously), respectively. RT-qPCR, Western blot, and immunofluorescence were used to detect the key factors' changes involved in promoting lung cancer proliferation, migration, and invasion.

The RT-qPCR analysis showed that the mRNA expressions of TRAF6, cFLIP, and CCL4 were significantly upregulated in the IL-1*β* group. Those genes were associated intimately with the IL-1*β*/NF-*κ*B signaling pathway. In contrast, cFLIP and CCL4 mRNA expression levels were downregulated in the FZQX group and the IL-1*β*+FZQX group compared with the IL-1*β* group. Furthermore, IL-1RA treatment could significantly attenuate XIAP and TRAF6 mRNA expression as compared with those in the control group. There were no significant differences in TRAF6, cFLIP, XIAP, and CCL4 mRNA expression levels among the IL-1RA group, the FZQX group, and the IL-1RA+FZQX group (as shown in Figures 5(b) and 5(c)).

At the protein expression level, Western blot and immunofluorescence analysis were carried out. Results indicated that key proteins in the IL-1*β*/NF-*κ*B signaling pathway were phosphorylated in the IL-1*β* group, and the proliferation and metastasis-associated proteins expression were significantly increased. In contrast, the expression levels of PCNA, Bcl-2, VEGFR, and MMP9 proteins in the FZQX group were significantly lower than those in the IL-1*β* group. There was no marked difference in protein expression between the FZQX group and the IL-RA group, which indicated that the inhibitory effect of the FZQX prescription was largely comparable with the IL-1 receptor antagonist (as shown in Figure 5(d)). These results were further confirmed by immunofluorescence staining (as shown in Figure 5(e)). These results demonstrated that the IL-1*β*/NF-*κ*B signaling pathway played a critical role in which the FZQX prescription suppresses the lung cancer progress via regulating MDSCs.

### 3.6. The FZQX Prescription Suppressed Lung Cancer Progression by Inhibiting the IL-1*β*/NF-*κ*B Pathway In Vivo

To further validate the potential molecular mechanism in vivo, a subcutaneous tumor xenograft model was established. Set the following groups including the control group (normal saline), the NF-*κ*B inhibitor group (SC75741 was injected intraperitoneally at the concentration of 15 mg/kg), and the NF-*κ*B inhibitor+F group (FZQX prescription was administered by gavage as described previously, whereas SC75741 was injected intraperitoneally). Tumor volumes of the three groups were observed throughout the duration of the experiment. At the endpoint of the experiment, the expression levels of tumor proliferation, migration, and invasion-related proteins in the IL-1*β*/NF-*κ*B pathway were detected by Western blot assay.

**Figure 6:**
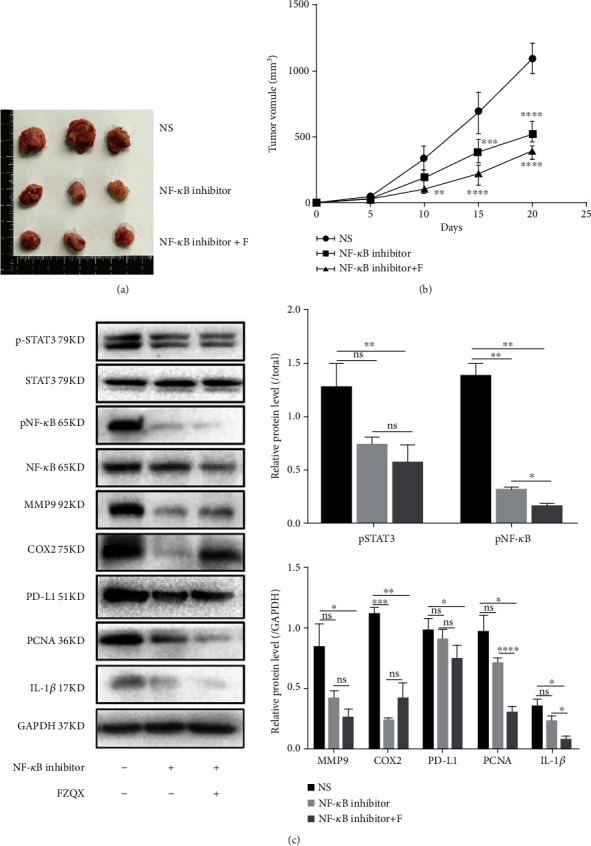
The FZQX prescription suppressed lung cancer progression by inhibiting the IL-1*β*/NF-*κ*B pathway in vivo. (a) Representative photographs of xenograft tumors in each group. (b) The volume of the xenograft tumors harvested from mice in each group. (c) Western blot detected the expression levels of tumor progression-related proteins and MDSCs related key proteins in the IL-1*β*/NF-*κ*B signaling pathway. NS: the normal saline group (control); NF-*κ*B inhibitor: mice were injected intraperitoneally with SC75741 (15 mg/kg); NF-*κ*B inhibitor + F: mice were given the FZQX prescription and injected intraperitoneal with SC75741 intraperitoneally.

Results showed that the tumor volume both in the NF-*κ*B inhibitor group and the NF-*κ*B inhibitor+F group underwent an obvious reduction. There was no significant difference between the two groups (as shown in Figures 6(a) and 6(b)). Western blot assay was used to detect the expression levels of tumor progression and MDSC activation-related factors in the IL-1*β*/NF-*κ*B pathway. Results indicated that the IL-1*β*/NF-*κ*B pathway was inhibited in the NF-*κ*B inhibitor group and the NF-*κ*B inhibitor+F group. The expression levels of tumor progression-related proteins (MMP9, PCNA, and IL-1*β*) and MDSCs' proliferation and activation-related proteins (COX2 and PDL1) were significantly downregulated (as shown in Figure 6(c)). Therefore, in vivo experiments also revealed that inhibiting the IL-1*β*/NF-*κ*B pathway was one of the important mechanisms for the FZQX prescription in preventing lung cancer progression via regulating MDSCs.

## 4. Discussion

As the tumor research progressed, increasing attention has been paid to the tumor immune microenvironment (TIME) in recent years [17]. It has been recognized that TIME is composed of cancer cells, lymphocytes, vascular endothelial cells, and stromal cells. Stromal cells, inflammatory cells, vascular system, extracellular matrix, and cytokines form a complex immunosuppressive network in the TIME [18], which plays a critical role in tumor immune escape. The levels of immune cells (macrophages, NK cells, CD_4_^+^T, CD_8_^+^T, Treg, MDSC) and cytokines (IL-1, TNF-*α*, and so on) in TIME show dynamic changes, which are affected by numerous factors [19]. Once the balance of the immune system interfered, it might favor immunological escape and contribute to the recurrence and metastasis after radical surgery.

As an essential complementary and alternative medicine, TCM has a unique advantage in tumor immunity regulation for its “holistic treatment concept” and has become a part of the comprehensive treatment of lung cancer [20]. Moreover, the early intervention for postoperative patients with early-stage lung cancer is a concrete embodiment of the concept of preventive philosophy in TCM. The FZQX prescription is an in-hospital preparation of the Shanghai Municipal Hospital of TCM. It is composed of twelve herbs, which have been applied for adjuvant therapy of lung cancer for about twenty years. Our previous retrospective cohort study demonstrated that the FZQX prescription could improve the mPFS of lung cancer patients, especially early-stage patients benefiting more indeed [21]. However, the precise clinical efficacy still needs scientific and objective evaluation. Therefore, to evaluate the clinical effect of the FZQX prescription in preventing recurrence and metastasis of early-stage lung adenocarcinoma cancer after surgery, we conducted a double-center, prospective cohort study. Results of the clinical trial showed that adjuvant therapy of the FZQX prescription improved the outcomes of postoperative patients with early-stage lung adenocarcinoma. The 3-year DFS rate in the treatment group was higher than that in the control group. Compared with the control group, there were significant improvements in the KPS score and the EORTC QLQ-LC43 scale after the FZQX prescription intervention. Furthermore, the level of negative immune cells (CD_8_^+^T, Treg, and MDSCs) in peripheral blood decreased, whereas that of the positive immune index (CD_4_^+^T/CD_8_^+^T) increased, which was close to that in the normal volunteer's group. These results confirmed that the FZQX prescription could effectively improve not only the quality of life but also the long-term survival rate in postoperative patients with early-stage lung adenocarcinoma, which may be related to the modulation of tumor immune cell proportions in the TIME.

In an efficacy-driven approach, clarifying the underlying molecular mechanism is also indispensable. Firstly, the MDSC clearance xenograft model was established with gemcitabine. Results showed that the FZQX prescription could effectively suppress tumor growth with lesser side effects, and the efficacy was similar to that of the MDSCs clearance group. To further validate the tumor-suppressive effect of the FZQX prescription and elucidate the role of MDSCs, a coculture system of MDSC/LLC (A549) was established through a conditional medium to simulate the TIME of lung cancer. Through a series of functional experiments such as colony-forming assay, wound healing assay, and Transwell invasion assay, it was confirmed that MDSCs could significantly promote the proliferation, migration, and invasion of LLC/A549 cells. On the contrary, the intervention of the FZQX prescription could effectively inhibit the above biological processes in the coculture system, which indicated that MDSCs play a crucial role in the intervention of lung cancer progression by the FZQX prescription.

MDSCs are a heterogeneous group of cells including myeloid progenitors and immature myeloid cells with immunosuppressive function [22]. In tumor-bearing mice, MDSCs were abnormally produced and recruited into the tumor microenvironment to establish an immunosuppressive environment that promoted tumor immune escape [23]. Previous studies have shown that various potential mechanisms were involved in the immunosuppressive function of MDSCs. However, the main immunosuppressive function of MDSCs is related to the high expression of arginase-1 (Arg-1) and ROS. Arg-1 can metabolize and deplete L-arginine, which is an essential substance for the proliferation and activation of T lymphocytes [24, 25]. ROS are a class of oxygenates with a more active chemical property converted from molecular oxygen, which includes superoxide anion (O_2_^−^), hydrogen peroxide (H_2_O_2_), hydroxyl radical (HO^−^), and nitrogen monoxide (NO) [26, 27]. ROS are involved in oxidative stress and play a regulatory role in the activation and proliferation of T cells in the TIME. High levels of ROS released from MDSCs could cause TCR nitration, inhibit T lymphocyte migration, and induce apoptosis of T lymphocytes and NK cells, thereby destroying the response-ability of immune cells [28]. Moreover, MDSCs can also upregulate the expression of PD-L1 and mediate tumor cell immune escape by interacting with its ligand PD-1 expressed by T lymphocytes [29]. Therefore, immunotherapy targeting MDSC clearance has drawn considerable attention recently. For instance, inhibiting the secretion of cytokines such as COX2, PGE2, VEGF, MMPs, and IL-6 can suppress the proliferation of MDSCs [30]. Downregulating the expression of TGF-*β*1 can reduce the levels of Arg-1 and iNOS, thus inhibiting the activation of MDSCs [31].

Inflammatory cytokines serve the role of communicators in the tumor microenvironment, which are together with immune cells in regulating the TIME and playing an important role in the occurrence and development of lung cancer [32]. Previous studies have shown that IL-1*β* secreted by macrophages, lung epithelial cells, and lung cancer cells contributed to the formation and maintenance of the tumor inflammatory environment [33]. On the other hand, the accumulation of IL-1*β* could form a positive feedback loop to promote lung cancer cells to further secrete cytokines such as VEGF and MMPs. The above changes in the tumor microenvironment could contribute to the development of lung cancer [34]. Furthermore, IL-1*β* could also induce the expression level of COX-2, act together with PGE2 to mediate the infiltration and activation of MDSCs, and play an immunosuppressive role. Clinical studies have also confirmed that elevated serum IL-1*β* level was closely related to poor prognosis in NSCLC [35].

Based on previous studies and cytokine levels quantified by ELISA, we focused on the inflammatory cytokine IL-1*β* in the tumor microenvironment to explore in-depth mechanisms. Therefore, the activator and the inhibitor of IL-1*β* were applied to the in vitro experiments. Results indicated that IL-1*β* played a critical role in MDSCs' mediated proliferation, migration, and metastasis of lung cancer cells, which was consistent with previous studies. The intervention of the FZQX prescription could effectively inhibit the progression of lung cancer by regulating MDSCs. Targeting IL-1*β* was one of the underlying mechanisms for the FZQX prescription in suppressing the proliferation and metastasis of lung cancer cells.

NF-*κ*B is a transcription factor involved in inflammatory regulation and immune response, which is closely related to tumorigenesis and apoptosis [36]. Inflammatory cytokine IL-1*β* could promote the phosphorylation and nuclear translocation of NF-*κ*B and activate the transcription of downstream tumor progression-related genes such as IL-1*β*, MMP9, and Bcl-2 to form another positive feedback loop, thus promoting the expression of antiapoptotic proteins and the proliferation and migration of tumor cells [37]. Studies have shown that the NF-*κ*B signaling pathway in lung epithelial cells was activated in NSCLC and accompanied by inflammatory factor infiltration [38]. Therefore, we applied the NF-*κ*B inhibitor for further verification. Experimental results showed that NF-*κ*B inhibitor could significantly downregulate the expression levels of tumor progression-related proteins and MDSCs' proliferation and activation-related proteins. No distinct difference was detected in protein expression levels between the NF-*κ*B inhibitor group and the NF-*κ*B inhibitor+F group. Hence, it could be considered that the IL-1*β*/NF-*κ*B signaling pathway was one of the key pathways of the FZQX prescription regulating MDSCs to inhibit lung cancer progression.

In summary, our clinical studies have confirmed that the FZQX prescription could effectively improve the 3-year DFS rate and life quality of postoperative patients with early-stage lung adenocarcinoma. After treatment with the FZQX prescription, the level of negative immune cells decreased, whereas the positive immune index increased. Based on the clinical findings, a series of in vivo and in vitro experiments confirmed the tumor-suppressive effect of the FZQX prescription and targeting MDSCs was a key step for FZQX prescription to exert its tumor-suppressive role. Finally, the activator and inhibitor of the pathway were applied to verify the regulation effect of the FZQX prescription on the IL-1*β*/NF-*κ*B signaling pathway. Results confirmed that the IL-1*β*/NF-*κ*B signaling pathway was one of the key pathways of the FZQX prescription in regulating MDSCs to inhibit lung cancer progression.

## 5. Conclusion

The FZQX prescription could effectively improve the 3-year DFS rate and life quality of postoperative patients with early-stage lung adenocarcinoma, which was accompanied by the increase of positive immune indexes and the decrease of negative immune indexes in peripheral blood. Experimental studies showed the FZQX prescription could inhibit the progression of lung cancer in vivo and in vitro by regulating the frequency and function of MDSCs. Further investigation confirmed that the IL-1*β*/NF-*κ*B signaling pathway was one of the key pathways for FZQX prescription involved in preventing recurrence and metastasis of lung cancer by regulating MDSCs.

## Figures and Tables

**Table 1:** The baseline clinical data of enrolled patients and healthy control population.

Characteristic	Control group (*n* = 241)	Treatment group (*n* = 224)	Healthy control group (n =110)	*χ* ^2^	*P*
Gender (%)				0.405	0.817
Male	85 (35.3)	76 (33.9)	35 (31.8)		
Female	156 (64.7)	148 (66.1)	75 (68.2)		
Age (%)				2.641	0.267
<60	110 (45.6)	119 (53.1)	53 (48.2)		
≥60	131 (54.4)	105 (46.9)	57 (51.8)		
Smoking (%)				0.158	0.924
No	220 (91.3)	204 (91.1)	99 (90.0)		
Yes	21 (8.7)	20 (8.9)	11 (10.0)		
TNM stage (%)				0.015	0.992
Tis	31 (12.9)	28 (12.5)	/		
IA	192 (79.7)	179 (79.9)	/		
IB	18 (7.5)	17 (7.6)	/		
Infiltration (%)				6.399	0.094
In situ	31 (12.9)	27 (12.1)	/		
Microinvasive	53 (22.0)	50 (22.3)	/		
Invasive	154 (63.9)	135 (60.3)	/		
Unknown	3 (1.2)	12 (5.4)	/		

No statistically significant difference was detected among three groups (*P* > 0.05). No statistically significant difference was detected between the control group and the treatment group (*P* > 0.05).

**Table 2:** Drug composition of the FZQX prescription.

Herb	Latin scientific name	Officinal part	Dosage (g)
Radix Astragali (Huang-Qi)	*Astragalus membranaceus* (Fisch.) Bunge.	Root	30
Atractylodis Macrocephalae Rhizoma (Bai-Zhu)	*Atractylis macrocephala* Koidz.	Rhizome	15
Poria (Bai-Fu-Ling)	*Poria cocos.*	Sclerotium	15
Glehniae Radix (Bei-Sha-Sheng)	*Glehnia littoralis* Fr.Schnidt.	Root	15
Radix Ophiopogonis (Mai-Dong)	*Ophiopogon japonicus* (L.f) Ker-Gawl.	Tuberous root	15
Codonopsis Radix (Dang-Shen)	*Codonopsis pilosula* (Franch.) Nannf.	Root	30
Bittersweet Herb (Shu-Yang-Quan)	*Solanum septemlobum* Bunge.	Herba	30
Prunellae spica (Xia-Ku-Cao)	*Prunella vulgaris* L.	Fruit cluster	30
Ostreae concha (Mu-Li)	*Ostreae gigas* Thunberg.	Shell	30
Chinese sage herb (Shi-Jian-Chuan)	*Salvia chinensis* Benth.	Herba	30
Herba Selaginellae Doederleinii (Shi-Shang-Bai)	*Selaginella doederleinii* Hieron.	Herba	30
Sargassum (Hai-Zao)	*Sargassum pallidum (Turn.)* C.Ag.	Frond	30

**Table:** (a) Comparison of EORTC QLQ-LC43 scale (functional areas) between treatment and control group before and after treatment

Group	*n*	EORTC QLQ-LC43 scale (functional areas)		*P* value
		Before treatment	After treatment	
Treatment group	224	31.99 ± 6.18	23.21 ± 7.66	<0.001
Control group	241	32.33 ± 10.02	27.10 ± 7.34	<0.001
*P* value		0.216	<0.001	

**Table:** (b) Comparison of EORTC QLQ-LC43 scale (general symptoms) between treatment and control group before and after treatment

Group	*n*	EORTC QLQ-LC43 scale (general symptoms)		*P* value
		Before treatment	After treatment	
Treatment group	224	27.93 ± 6.91	21.15 ± 5.49	<0.001
Control group	241	26.98 ± 7.08	20.83 ± 5.29	<0.001
*P* value		0.138	0.565	

**Table:** (c) Comparison of EORTC QLQ-LC43 scale (special symptoms) between treatment and control group before and after treatment

Group	*n*	EORTC QLQ-LC43 scale (special symptoms)		*P* value
		Before treatment	After treatment	
Treatment group	224	33.45 ± 9.46	22.15 ± 6.91	<0.001
Control group	241	32.23 ± 9.22	26.44 ± 7.75	<0.001
*P* value		0.329	<0.001	

**Table:** (d) Comparison of EORTC QLQ-LC43 scale (general health) between treatment and control group before and after treatment

Group	*n*	EORTC QLQ-LC43 scale (general health)		*P* value
		Before treatment	After treatment	
Treatment group	224	8.20 ± 2.81	10.87 ± 1.83	<0.001
Control group	241	8.70 ± 2.93	10.09 ± 2.65	<0.001
*P* value		0.158	0.002	

**Table:** (e) Comparison of EORTC QLQ-LC43 scale (economic difficulties) between treatment and control group before and after treatment

Group	*n*	EORTC QLQ-LC43 scale (economic difficulties)		*P* value
		Before treatment	After treatment	
Treatment group	224	1.09 ± 0.31	1.09 ± 0.30	0.317
Control group	241	1.07 ± 0.25	1.09 ± 0.28	0.411
*P* value		0.934	0.941	

**Table 4:** Comparison of KPS between treatment and control group before and after treatment.

Group	*n*	KPS		*P* value
		Before treatment	After treatment	
Treatment group	224	79.29 ± 9.25	87.14 ± 5.98	<0.001
Control group	241	79.13 ± 8.04	83.11 ± 9.03	<0.001
*P* value		5.191	<0.001	

**Table 5:** Comparison of life quality improvement between treatment and control group before and after treatment (*n*, %).

Group		Life quality improvement			*P* value
	*n*	Improvement	Stability	Deterioration	
Treatment group	224	131 (58.5)	81 (36.2)	12 (5.4)	<0.001
Control group	241	100 (41.5)	108 (44.8)	33 (13.7)	

**Table:** (a) Comparison of T-cell subsets among treatment, control group, and healthy volunteers' group

Group	*n*	CD_4_^+^T		CD_8_^+^T		CD_4_^+^/CD_8_^+^	
		Before treatment	After treatment	Before treatment	After treatment	Before treatment	After treatment
Treatment group	224	37.14 ± 8.58	36.98 ± 8.72^△^	24.38 ± 8.42	23.52 ± 7.99^∗∗^	1.81 ± 1.53	2.14 ± 5.03^∗∗^
Control group	241	38.57 ± 8.95	38.37 ± 9.93^△^	22.85 ± 8.33	23.03 ± 8.02^△^	1.96 ± 1.09	1.94 ± 1.08^△^
Health group	110	40.72 ± 14.13		21.86 ± 9.33		2.19 ± 1.11	
*P* value		0.081	0.116	0.080	0.634	0.007	0.247

^△^No statistically significant difference was seen before and after treatment (*P* > 0.05). ^∗∗^Statistically significant difference was seen before and after treatment (*P* < 0.05).

**Table:** (b) Comparison of MDSCs and Treg among treatment, control group, and healthy volunteers' group

Group	*n*	MDSCs		Treg	
		Before treatment	After treatment	Before treatment	After treatment
Treatment group	224	7.11 ± 6.65	5.89 ± 5.62^∗∗^	2.28 ± 2.52	1.81 ± 2.15^∗∗^
Control group	241	7.02 ± 4.95	7.54 ± 5.38^△^	2.20 ± 1.30	2.41 ± 1.95^△^
Health group	110		1.06 ± 1.99		0.74 ± 0.82
*P* value		0.305	0.001	0.055	<0.001

^△^No statistically significant differences were seen before and after treatment (*P* > 0.05). ^∗∗^Statistically significant differences were seen before and after treatment (*P* < 0.05).

**Table 7:** Comparison of disease-free survival rates between treatment and control group.

Group	*n*	Events	1-year DFS rate (%)	2-year DFS rate (%)	3-year DFS rate (%)	*χ* ^2^	*P* value
Treatment group	224	7	99.1	97.0	93.7	4.751	0.029
Control group	241	17	97.6	91.7	87.5		

**Table 8:** Inhibitory effects of each group on xenograft tumors of mice.

Group	Tumor-free weight	Tumor volume	Tumor suppression rate
NS	25.14 ± 0.75	1300.37 ± 354.48	—
FZQX	25.90 ± 1.40	706.69 ± 134.35^∗∗^	45.65%
GEM	21.89 ± 1.92^∗^	378.30 ± 93.61^∗∗∗^	70.91%
FZQX+GEM	25.65 ± 1.03	290.93 ± 103.43^∗∗∗^	77.63%

^∗^ *P* < 0.05 compared with the NS group; ^∗∗^*P* < 0.01 compared with the NS group; ^∗∗∗^*P* < 0.001 compared with the NS group.

## Data Availability

The datasets generated for this study are available on request to the corresponding author.

## Abbreviations

FZQX: Fu-Zheng-Qu-Xie
MDSCs: Myeloid-derived suppressor cells
LLC: Lewis lung cancer cells
TIME: Tumor immune microenvironment
NSCLC: Non-small-cell lung cancer
ChiCTR: Chinese Clinical Trial Registry
CT: Computed tomography
MRI: Magnetic resonance imaging
KPS: Karnofsky Performance Score
QoL: Quality of life
DFS: Disease-free survival
PFS: Progression-free survival
NCCN: National comprehensive cancer network
UPLC: Ultraperformance liquid chromatography
TCM: Traditional Chinese medicine
DMEM: Dulbecco's modification of Eagle's medium Dulbecco
FBS: Fetal bovine serum
RIPA: Radio immunoprecipitation assay
RPM: Revolutions per minute
IL: Interleukin
TGF-*β*: Transforming growth factor-*β*
PBS: Phosphate buffer saline
SPF: Specific pathogen free
PVDF: Polyvinylidene fluoride
ECL: Enhanced chemiluminescence
BSA: Bovine serum albumin
ANOVA: Analysis of variance
GEM: Gemcitabine
MMP: Matrix metallopeptidase
PTGER: Prostaglandin E receptor
STAT3: Signal transducer and activator of transcription 3
TRAF: TNF receptor associated factor
cFLIP: Cellular FLICE inhibitory protein
XIAP: X-linked inhibitor of apoptosis protein
PCNA: Proliferating cell nuclear antigen
VEGFR: Vascular endothelial growth factor receptor
PFA: Paraformaldehyde.

## Supplementary Materials

Supplementary Materials Supplemental Methods. Figure S1: the flow chart describing enrollment, screening, grouping, and follow-up results of the prospective cohort study. Figure S2: base peak chromatogram of the FZQX prescription in positive mode (a) and negative mode (b). Figure S3: MDSC induction and characterization by flow cytometry and RT-qPCR. (a) The cell morphological changes during the induction of BM-MDSCs were visualized under a light microscope (magnification, ×400); (b) the expression levels of BM-MDSCs before and after induction detected by flow cytometry; (c) the mRNA expression levels of key immunosuppressive factors of MDSCs before and after induction; (d) the mRNA expression levels of MDSCs related cytokines before and after induction. Figure S4: the establishment procedure of conditioned medium. Figure S5: cell viability was detected by CCK-8 assay, and IC50 values were calculated using nonlinear regression. Table S1: information of 71 compounds identified from the FZQX prescription by UPLC/QTOF MS. TableS2: list of primer sequences used in RT-qPCR. TableS3: list of primary antibodies used in Western blot, immunofluorescence, and immunohistochemistry.
