# Supplementary material for: Chinese Herbal Prescription Fu-Zheng-Qu-Xie Prevents Recurrence and Metastasis of Postoperative Early-Stage Lung Adenocarcinoma: A Prospective Cohort Study Followed with Potential Mechanism Exploration
Source: Oxid Med Cell Longev. 2021 May 12;2021:6673828. doi: 10.1155/2021/6673828 (PMC8133853; doi:10.1155/2021/6673828)
Supplement: Supplementary Materials — Supplemental Methods. Figure S1: the flow chart describing enrollment, screening, grouping, and follow-up results of the prospective cohort study. Figure S2: base peak chromatogram of the FZQX prescription in positive mode (a) and negative mode (b). Figure S3: MDSC induction and characterization by flow cytometry and RT-qPCR. (a) The cell morphological changes during the induction of BM-MDSCs were visualized under a light microscope (magnification, ×400); (b) the expression levels of BM-MDSCs before and after induction detected by flow cytometry; (c) the mRNA expression levels of key immunosuppressive factors of MDSCs before and after induction; (d) the mRNA expression levels of MDSCs related cytokines before and after induction. Figure S4: the establishment procedure of conditioned medium. Figure S5: cell viability was detected by CCK-8 assay, and IC50 values were calculated using nonlinear regression. Table S1: information of 71 compounds identified from the FZQX prescription by UPLC/QTOF MS. TableS2: list of primer sequences used in RT-qPCR. TableS3: list of primary antibodies used in Western blot, immunofluorescence, and immunohistochemistry. [file 6673828.f1.docx]

**Supplemental Methods**

**The inclusion criteria were as followed:**

1. The postoperative pathological stages of lung adenocarcinoma were Tis, IA1-3, and IB, and patients received no adjuvant therapy after radical surgery.
2. Patient ages ranged from 18-90 years old.
3. Biochemical and hematological blood were all within normal ranges.
4. A lung adenocarcinoma diagnosis without other synchronous malignancies or serious systemic diseases.
5. Patients have good compliance and can cooperate with the researcher’s follow-up study.

**The exclusion criteria were as followed:**

1. Patients with recurrence and metastasis have occurred.
2. Patients receiving the other CHM, chemotherapy, targeted therapy, or any other interventions now or in the past 3 months.
3. Patients with mental illness or were incapacitated.
4. Pregnant, lactating women or other women have a reproductive requirement were excluded.
5. Patients combined with serious diseases including heart, liver, kidney failure, neuropsychiatric disorders, severe infection, etc.
6. Patients with drug alcohol abuse/dependence.
7. Patients with poor adherence according to the judgment of the researchers.


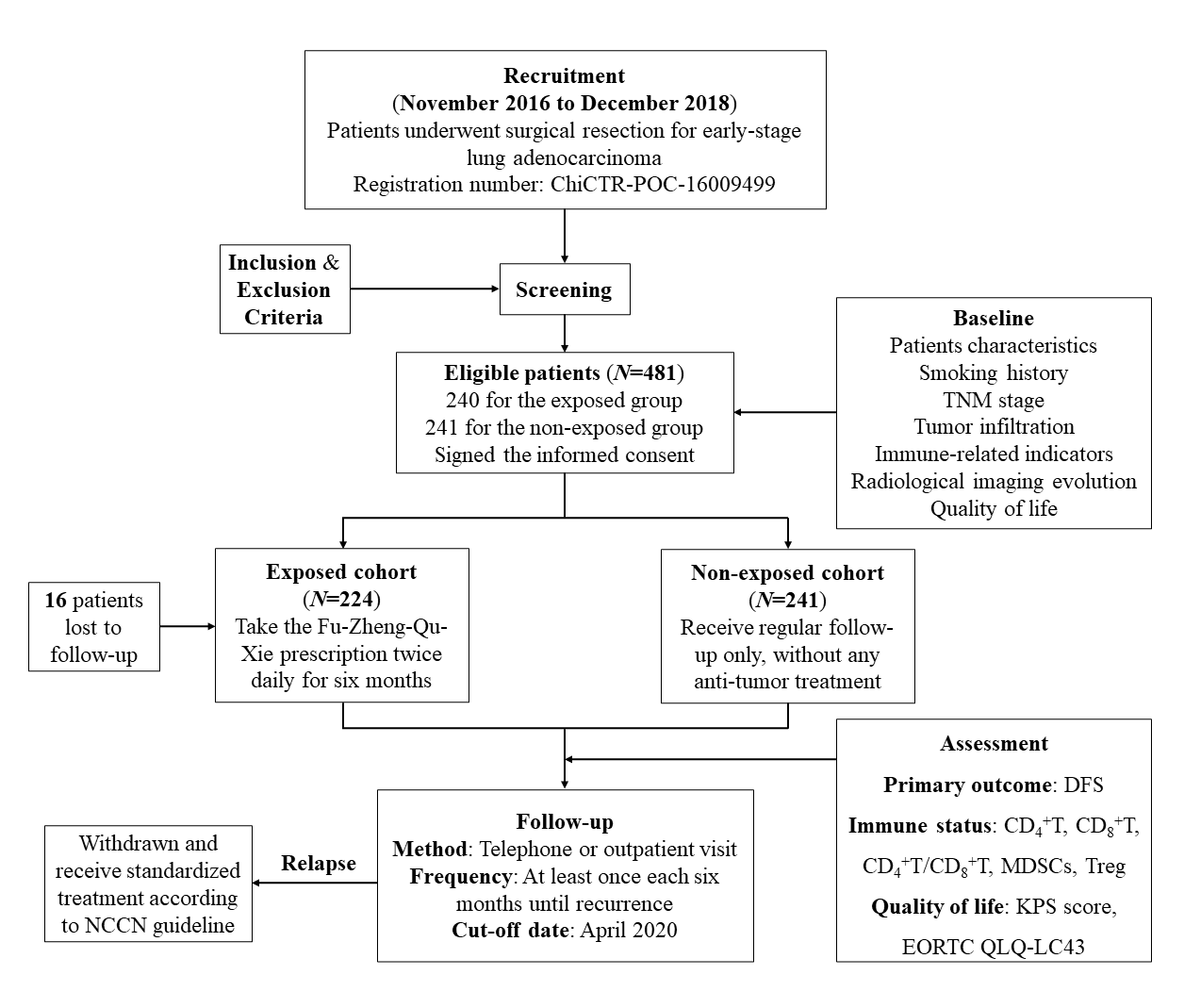


**Figure S1:** The flow chart describing enrollment, screening, grouping, and follow-up results of the prospective cohort study.


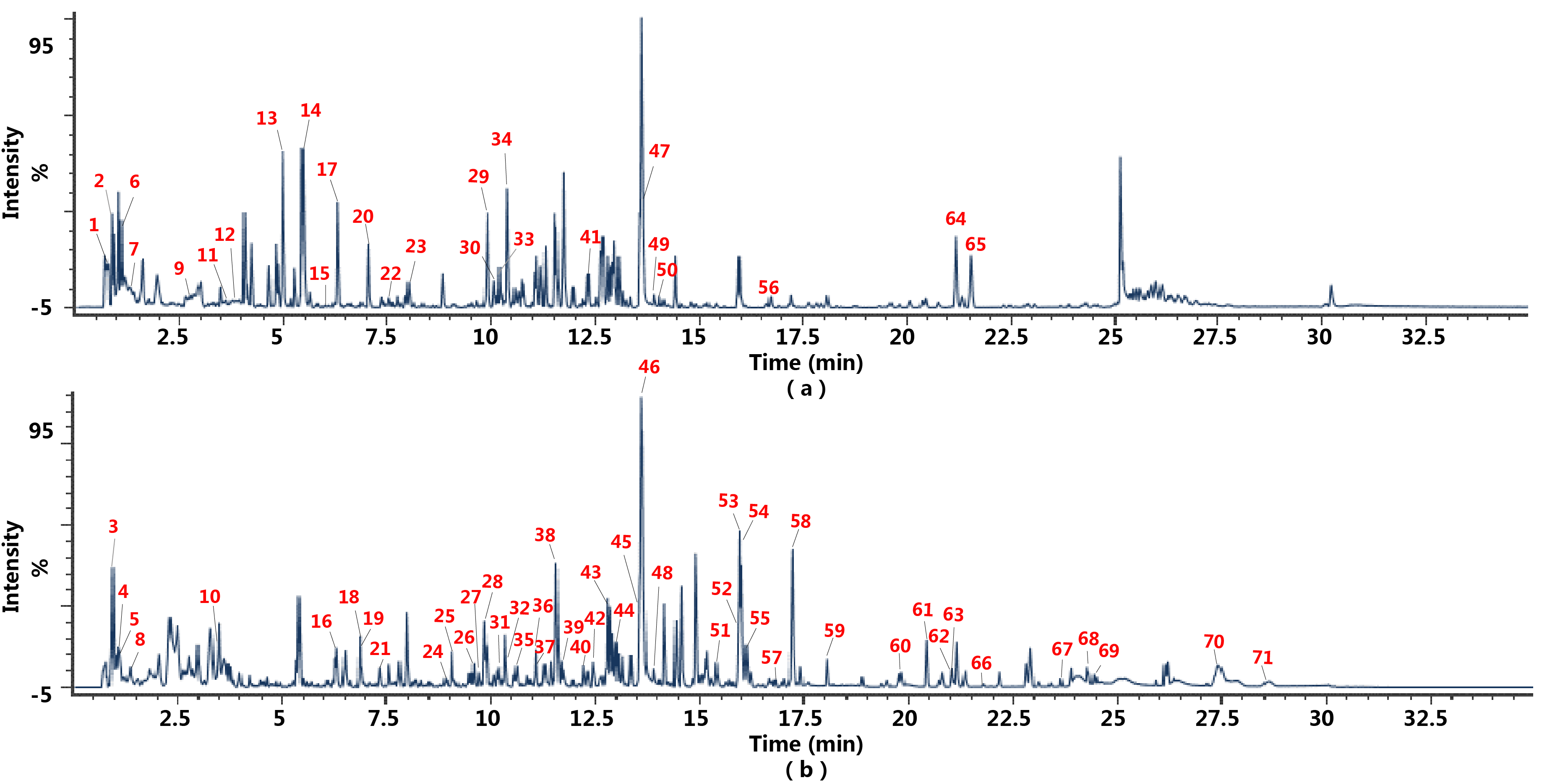


**Figure S2:** Base peak chromatogram of the FZQX prescription in positive mode (a) and negative mode (b).


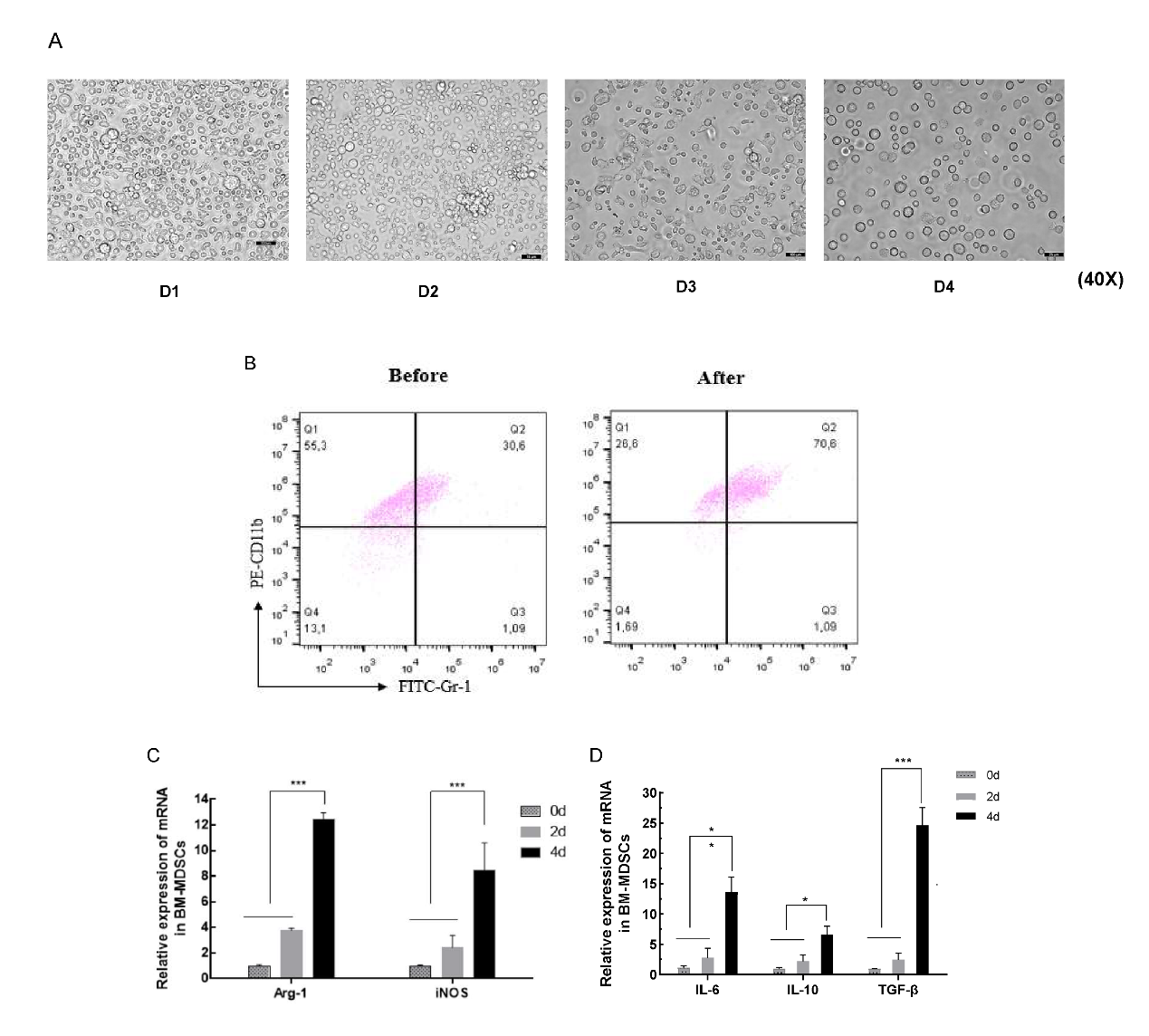


**Figure S3:** MDSCs induction and characterization by flow cytometry and RT-PCR. **(A)** The cell morphological changes during the induction of BM-MDSCs were visualized under a light microscope (magnification, ×400); **(B)** The expression levels of BM-MDSCs before and after induction detected by flow cytometry; **(C)** The mRNA expression levels of key immunosuppressive factors of MDSCs before and after induction; **(D)** The mRNA expression levels of MDSCs related cytokines before and after induction.

**
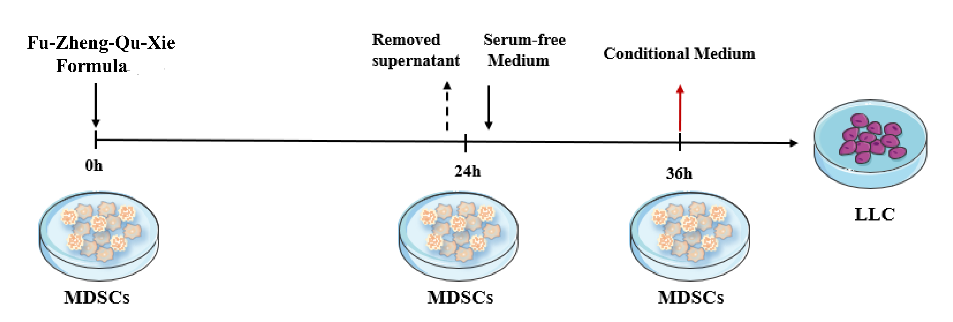
**

**Figure S4:** The establishment procedure of conditioned medium.


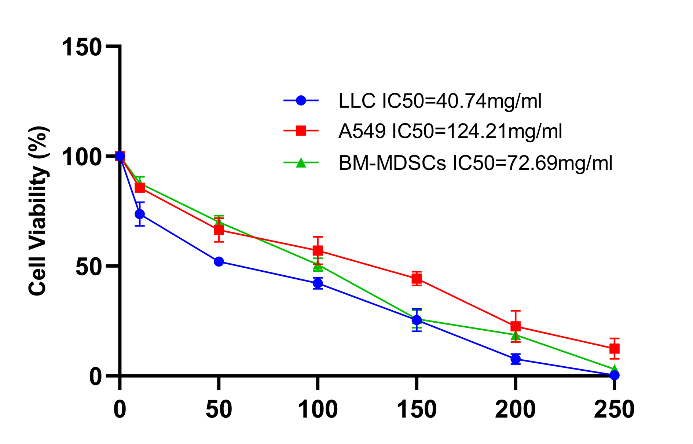


**Figure S5**: Cell viability was detected by CCK-8 assay and IC50 values were calculated using non-linear regression.

**Table S1: Information of 71 compounds identified from the FZQX prescription by UPLC/QTOF MS**

| **No.** | **Identification status** | **Observed m/z** | **Mass error (mDa)** | **Observed RT (min)** | **Theoretical Fragments Found** | **Adducts** | **TCM** |
| --- | --- | --- | --- | --- | --- | --- | --- |
| 1 | 3-methoxy-4-hydroxybenzoic acid | 151.0346 | -4.3 | 0.7 | 0 | -H20+H | DS/MD/HZ |
| 2 | arginine | 175.1185 | -0.4 | 0.87 | 2 | +H, +Na | BSS/BZ/DS/HQ |
| 3 | pentose | 195.0509 | -0.1 | 0.95 | 1 | +HCOO, -H2O+HCOO | HQ/BZ/DS/BFL/BSS/SJC/SSB/MD/SYQ/HZ/XKC |
| 4 | hesperidin | 609.1873 | 4.9 | 1.07 | 24 | -H | SJC/SYQ |
| 5 | coreline C | 533.1713 | 4.8 | 1.08 | 12 | -H2O-H | DS |
| 6 | proline | 138.0543 | 1.7 | 1.08 | 1 | +Na, +H | BZ/DS/HQ/MD/SYQ |
| 7 | methyl ophiopogon flavanone A | 365.1039 | 4.3 | 1.3 | 0 | +Na | BZ |
| 8 | shikimic acid | 173.0455 | 0 | 1.56 | 2 | -H, -H2O-H | SSB |
| 9 | glutamate | 130.0493 | -0.5 | 2.76 | 1 | -H20+H | BSS/DS/MD/SJC/SSB/SYQ |
| 10 | *β* -uridine | 243.0622 | -0.1 | 3.4 | 3 | -H | BZ |
| 11 | methyl ophiopogon flavanone A isomer | 325.1117 | 4.6 | 3.51 | 1 | -H20+H | MD |
| 12 | methyl ophiopogon flavanone A isomer | 325.1116 | 4.5 | 3.75 | 1 | -H20+H | BZ |
| 13 | hordenine-O-α-L-rhamnoside | 312.1799 | -0.6 | 4.98 | 7 | +H | SSB |
| 14 | codonopsis | 268.1543 | 0 | 5.45 | 4 | +H | DS |
| 15 | secoisolariciresinol | 367.1487 | -2.9 | 6.11 | 0 | -H2O+Na | BSS/DS |
| 16 | chlorogenic acid | 353.0881 | 0.3 | 6.26 | 2 | -H | XKC |
| 17 | tryptophan | 205.0967 | -0.4 | 6.29 | 9 | +H | BSS/DS/HQ/MD |
| 18 | vitamin B1 | 299.0777 | 3.8 | 6.88 | 1 | -H | HQ |
| 19 | macrophyllin | 137.0248 | 0.4 | 6.89 | 2 | -H2O-H | SYQ/HQ |
| 20 | falcarindiol | 265.1547 | -1.6 | 7.03 | 5 | -H2O+Na | SYQ |
| 21 | chlorogenic acid | 353.0872 | -0.6 | 7.33 | 4 | -H | XKC/BZ |
| 22 | swertimarin | 357.1173 | -0.7 | 7.53 | 7 | -H20+H | SJC/SYQ |
| 23 | *cis*-caffeic acid | 163.0385 | -0.5 | 8 | 2 | -H20+H, +H | SJC/SYQ/XKC |
| 24 | codonopsis glycoside I | 677.2293 | -0.5 | 8.94 | 3 | -H | DS |
| 25 | salvianolic acid D | 463.0915 | 3.3 | 9.06 | 6 | +HCOO, -H | XKC |
| 26 | poriol | 331.0819 | -0.4 | 9.59 | 5 | +HCOO | SJC |
| 27 | rutin isomer | 609.1461 | 0 | 9.76 | 1 | -H | SYQ |
| 28 | rutin isomer | 609.1457 | -0.4 | 9.85 | 7 | -H | SJC/SYQ/XKC |
| 29 | 3'-methoxy-5'-hydroxyisoflavone-7-O-β-D-glucoside | 447.128 | -0.5 | 9.9 | 6 | +H, +Na | HQ |
| 30 | 6-methoxy-7-hydroxycoumarin | 193.049 | -0.5 | 10.14 | 0 | +H | BSS/SYQ |
| 31 | chlorogenic acid | 353.0876 | -0.2 | 10.16 | 3 | -H | SSB |
| 32 | salvianolic acid D | 463.0878 | -0.4 | 10.19 | 9 | +HCOO | SYQ |
| 33 | isoquercetin | 465.1018 | -0.9 | 10.2 | 5 | +H | SJC/XKC |
| 34 | *cis*-caffeic acid | 163.0385 | -0.5 | 10.33 | 3 | -H20+H | XKC |
| 35 | selaginin B | 521.2024 | -0.5 | 10.61 | 10 | -H2O-H, -H2O+HCOO | SSB |
| 36 | calycosin | 283.0609 | -0.3 | 11.28 | 7 | -H | HQ |
| 37 | salvianolic acid B isomer | 717.1436 | -2.5 | 11.32 | 14 | -H | SYQ |
| 38 | rosmarinic acid | 359.0767 | -0.6 | 11.56 | 21 | -H | SYQ |
| 39 | salvianolic acid A | 493.1125 | -1.6 | 11.67 | 11 | -H | SJC |
| 40 | salvianolic acid B isomer | 717.144 | -2.1 | 12.21 | 14 | -H | SJC |
| 41 | 4'-Hydroxyimperatorin-4'-O-β-D-pyranoglucoside | 431.1327 | -1 | 12.32 | 10 | -H20+H, -H2O+Na | HQ |
| 42 | sericoside | 711.3952 | -0.9 | 12.44 | 4 | +HCOO | SJC/SYQ |
| 43 | borneol-2-O-β-D-celery-(1_6) -β-D-glucoside | 493.2278 | -1.2 | 12.73 | 0 | +HCOO | MD |
| 44 | 9,10-dimethoxy sandalwood-3-O-β-D-glucoside | 507.1511 | 0.3 | 13.05 | 30 | +HCOO | HQ |
| 45 | poriol | 267.0663 | 0.1 | 13.55 | 4 | -H2O-H | HQ |
| 46 | 14-hydroxy sprengerinin C isomer | 915.4586 | -0.9 | 13.61 | 51 | +HCOO, -H2O+HCOO | XKC |
| 47 | calycosin | 285.0752 | -0.6 | 13.65 | 2 | +H | HQ |
| 48 | Ophiopogon japonicus saponins E | 769.4003 | -1.3 | 13.89 | 6 | +HCOO | XKC |
| 49 | lycium barbarum C | 964.4177 | 0.2 | 13.92 | 25 | +Na | SSB/SYQ |
| 50 | quercetin-3-methyl ether | 339.0484 | 0.9 | 14.14 | 3 | +Na, -H2O+Na | XKC |
| 51 | astragaloside A | 695.4006 | -0.6 | 15.37 | 5 | +HCOO | SJC |
| 52 | dehydrotumulosic acid | 529.3527 | -0.8 | 15.9 | 1 | +HCOO | BFL |
| 53 | 14-sydroxy sprengerinin C isomer | 897.4459 | -3 | 15.96 | 16 | -H2O+HCOO | XKC |
| 54 | tianshic acidisomer | 329.2329 | -0.4 | 16 | 7 | -H | SJC/SSB/SYQ/XKC/BFL/DS/HQ |
| 55 | tianshic acidisomer | 329.2326 | -0.7 | 16.11 | 4 | -H | XKC/BFL/DS |
| 56 | poriol | 269.0801 | -0.7 | 16.72 | 1 | -H20+H | HQ |
| 57 | astragaloside IV | 829.4565 | -2.6 | 16.95 | 4 | +HCOO | HQ |
| 58 | tianshic acidisomer | 329.2327 | -0.7 | 17.21 | 5 | -H | XKC |
| 59 | astragaloside II | 871.4683 | -1.4 | 18.05 | 2 | +HCOO | HQ |
| 60 | poricoic acid DM | 555.3312 | -1.5 | 19.84 | 1 | -H2O+HCOO | SYQ/DS/HQ |
| 61 | isoastragaloside I | 913.4796 | -0.7 | 20.43 | 3 | +HCOO | HQ |
| 62 | asparagin B | 749.4108 | -1 | 21.02 | 1 | -H2O+HCOO | SYQ |
| 63 | 25-hydroxyporicoic acid H | 497.3265 | -0.8 | 21.09 | 1 | -H2O-H | BFL |
| 64 | 3β-hydroxyapatrone isomer | 215.1422 | -0.9 | 21.17 | 23 | -H20+H, +H | SJC |
| 65 | 3β-hydroxyapatrone isomer | 215.1423 | -0.8 | 21.54 | 27 | -H20+H, +H | SJC |
| 66 | sprengerinin C | 899.4641 | -0.5 | 21.64 | 1 | +HCOO | MD |
| 67 | asparagin B' | 941.4754 | 0.3 | 23.65 | 0 | +HCOO | MD |
| 68 | 26-hydroxyporicoic acid DM | 589.3379 | -0.3 | 24.27 | 3 | +HCOO | XKC |
| 69 | 3-O-acetyl-16a-hydroxy-dehydrotrametenolic acid | 539.335 | -2.8 | 24.43 | 1 | -H2O+HCOO | SYQ |
| 70 | tanshinone diphenol | 297.1532 | 3.6 | 27.43 | 1 | -H | HZ/ML/SYQ |
| 71 | ergosterol | 423.3272 | 0.3 | 28.98 | 1 | -H2O+HCOO | BFL |

Note: HQ(Huang-Qi): *Astragalus membranaceus* (Fisch.) Bge. Var. *mongholicus* (Bge.) Hsiao; BZ(Bai-Zhu): *Atractylodes macrocephala* Koidz.; MD(Mai-Dong): *Ophiopogon Japonicus* (L.f) Ker-Gawl.; BSS(Bei-Sha-Shen): *Glehnia littoralis* Fr. Schnidt; Poria (Bai-Fu-Ling): Poria *cocos* (Schw.) Wolf.**;** Codonopsis Radix (Dang-Shen): *Codonopsis pilosula* (Franch.) Nannf. & *Codonopsis pilosula* Nannf. *var. Modesta* (Nannf.) L. T. Shen & *Codonopsis tangshen* Oliv.; SSB(Shi-Shang-Bai): *Selaginella doederleinii* Hieron.; SJC(Shi-Jian-Chuan): *Salvia chinensis* Benth.; SYQ(Shu-Yang-Quan): *Solanum septemlobum* Bunge; XKC(Xia-Ku-Cao): *Prunella vulgaris* L.; HZ(Hai-Zao): *Sargassum* *pallidum*(Turn.) C.Ag. & Sargassum *fusiforme* (Harv.) Setch. and ML(Mu-Li): *Ostreae gigas* Thunberg.&*Ostrea talienwhanensis* Crosse.&*Ostrea rivularis* Gould

**TableS2 List of primer sequences used in RT-qPCR**

| Gene Name | Forward Primer | Reverse Primer |
| --- | --- | --- |
| *Arg-1* | TGCTCACACTGACATCAACACTCC | TCTACGTCTCGCAAGCCAATGTAC |
| *iNOS* | TGCCACGGACGAGACGGATAG | CTCTTCAAGCACCTCCAGGAACG |
| *NO* | GTCAGAAGATGTCCGCACCAAGG | TGTTCACCTCCTCCAGCCTGTC |
| *CCL4* | CTTGCTCGTGGCTGCCTTCTG | AGCTGCCGGGAGGTGTAAGAG |
| *TRAF6* | TCCAGCCAGTCGTCCAGTGAC | CAGAGCCACTCACGCTGTCATC |
| *c-FLIP* | TGGCAGAATATGAAGCACGGATCG | TGAGCCCTCCTCCACAGTGAAAG |
| *XIAP* | TGGCAGAATATGAAGCACGGATCG | TGAGCCCTCCTCCACAGTGAAAG |
| *IL-6* | ACTTCCATCCAGTTGCCTTCTTGG | TTAAGCCTCCGACTTGTGAAGTGG |
| *IL-10* | CTGCTATGCTGCCTGCTCTTACTG | ATGTGGCTCTGGCCGACTGG |
| *TGF-β* | AACACTTCGGCTCCTCAAGACATC | CCAGAAGCTGAACCTACCATCGG |
| *GAPDH* | GGAGCGAGATCCCTCCAAAAT | GGCTGTTGTCATACTTCTCATGG |

**TableS3 List of primary antibodies used in Western blot and immunofluorescence**

| Antigen | host | Dilution | Source |
| --- | --- | --- | --- |
| VEGFR2 | Rabbit | 1:1000 | Cell Signaling Technology 9698 |
| MMP9 | Rabbit | 1:1000 | Cell Signaling Technology 13667 |
| PTGER2 | Rabbit | 1:20000 | Abcam 167171 |
| Bcl-2 | Rabbit | 1:1000 | Cell Signaling Technology 4223 |
| IL-1β | Rabbit | 1:1000 | Cell Signaling Technology 83186 |
| β-actin | Rabbit | 1:1000 | Cell Signaling Technology 4970 |
| p-STAT3 | Rabbit | 1:2000 | Cell Signaling Technology 9145 |
| STAT3 | Rabbit | 1:1000 | Cell Signaling Technology 12640 |
| pNF-κB | Rabbit | 1:1000 | Cell Signaling Technology 3033 |
| NF-κB | Rabbit | 1:1000 | Cell Signaling Technology 8242 |
| GAPDH | Rabbit | 1:1000 | Cell Signaling Technology 5174 |
| PCNA | Rabbit | 1:1000 | Cell Signaling Technology 13110 |
| COX-2 | Rabbit | 1:1000 | Cell Signaling Technology 12282 |
| PD-L1 | Rabbit | 1:1000 | Cell Signaling Technology 13684 |
| Ki-67 | Rabbit | 1:5000 | Abcam 92742 |
